# Presentation of B-cell lymphoma in childhood and adolescence: a systematic review and meta-analysis

**DOI:** 10.1186/s12885-024-12372-w

**Published:** 2024-06-11

**Authors:** Defne Saatci, C. Zhu, A. Harnden, J. Hippisley-Cox

**Affiliations:** 1 Nuffield Department of Primary Care Health Sciences, University of Oxford, Woodstock Road, Oxford, OX2 6GG UK; 2 UCL Cancer Institute, University College London, London, UK

**Keywords:** “childhood lymphoma”, “adolescent lymphoma”, “clinical presentation”, “symptom prevalence”, “meta-analysis of proportions”

## Abstract

**Background:**

The diagnosis of B-cell lymphoma, one of the commonest cancers seen in childhood and adolescence, is challenging. There is a crucial need to identify and delineate the prevalence of associated symptoms in order to improve early diagnosis.

**Aims:**

To identify clinical presentations associated with childhood and adolescent B-cell lymphomas and estimate symptom prevalence.

**Methods:**

A systematic review of observational studies and meta-analysis of proportions was carried out. Medline and EMBASE were systematically searched, with no language restrictions, from inception to 1st August 2022. Observational studies with at least 10 participants, exploring clinical presentations of any childhood and adolescent lymphoma, were selected. Proportions from each study were inputted to determine the weighted average (pooled) proportion, through random-effects meta-analysis.

**Results:**

Studies reported on symptoms, signs and presentation sites at diagnosis of 12,207 children and adolescents up to the age of 20. Hodgkin’s lymphoma most frequently presented with adenopathy in the head-and-neck region (79% [95% CI 58%-91%]), whilst non-Hodgkin’s lymphoma presented abdominally (55% [95% CI 43%-68%]). Symptoms associated with lymphoma included cervical lymphadenopathy (48% [95% CI 20%-77%]), peripheral lymphadenopathy (51% [95% CI 37%-66%]), B-symptoms (40% [95% CI 34%-44%]), fever (43% [95% CI 34%-54%]), abdominal mass (46% [95% CI 29%-64%]), weight loss (53% [95% CI 39%-66%]), head-and-neck mass (21% [95% CI 6%-47%]), organomegaly (29% [95% CI 23%-37%]), night sweats (19% [95% CI 10%-32%]), abdominal pain (28% [95% CI 15%-47%]), bone pain (17% [95% CI 10%-28%]) and abnormal neurology (11% [95% CI 3%-28%]).

**Conclusion:**

This systematic review and meta-analysis of proportions provides insight into the heterogeneous clinical presentations of B-cell lymphoma in childhood and adolescence and provides estimates of symptom prevalence. This information is likely to increase public and clinical awareness of lymphoma presentations and aid earlier diagnosis. This review further highlights the lack of studies exploring childhood and adolescent lymphoma presentations in primary care, where patients are likely to present at the earliest stages of their disease.

**Supplementary Information:**

The online version contains supplementary material available at 10.1186/s12885-024-12372-w.

## Introduction

Childhood and adolescent cancers are uncommon, with an estimated global incidence rate of 156 per million [1]. Despite this low incidence, cancer is the commonest cause of death and disability in children and adolescents across the world, carrying vast human, socioeconomic and healthcare costs [2]. Early recognition of cancer plays an important role in reducing this long-term burden [3]. One of the biggest challenges to early recognition is the non-specific presentation of cancers in this age group, mimicking symptoms and signs associated with self-limiting diseases in childhood and adolescence.

Childhood and adolescent B-cell lymphomas are one of the commonest cancers in this age group and can be broadly categorised into Hodgkin’s lymphoma and B-cell non-Hodgkin’s lymphoma (e.g., mature B-cell neoplasms) [4]. Hodgkin’s lymphoma is frequently encountered in adolescence [5] whilst B-cell non-Hodgkin’s lymphoma across childhood and adolescence [6]. Lymphomas present heterogeneously across a range of sites on the body and are associated with a varied number of non-specific symptoms and signs [7]. Examples include Burkitt’s lymphoma which presents more commonly with facial or abdominal swelling and Hodgkin’s lymphoma with painless lymphadenopathy [7] and non-specific symptoms such as fever, weight loss and night sweats (i.e. B-symptoms). Such non-specific symptoms, coupled with the relative rarity of lymphoma, make prompt recognition by healthcare professionals particularly challenging. Lymphomas have one of the most protracted diagnostic intervals within cancers in this age group and are more frequently associated with advanced stages at diagnosis [8].

Several observational studies to date have explored clinical presentations of childhood and adolescent cancers to advance clinical knowledge and awareness in this area [9–12]. However, these studies are limited by sample size, with the majority based on single-centre data of a few hundred patients [13]. Additionally, as cancer in this age group is rare, conducting large observational, multi-centre cohorts have been limited by high costs. To overcome this challenge, the clinical presentations of other childhood cancers, such as leukaemias and central nervous system tumours, have been summarised in comprehensive literature reviews, providing the most up-to-date evidence for associated symptoms and signs [14, 15]. These reviews have highlighted the wide range of symptoms associated with childhood cancers, increased public and clinical awareness, and informed clinical guidance. Despite significant challenges in early diagnosis, there have been no literature reviews summarising clinical presentations or the relative importance of an individual symptom in a lymphoma diagnosis in children and adolescents to date. Accordingly, we have systematically reviewed all existing evidence and carried out a meta-analysis of proportions to advance knowledge of clinical presentations of B-cell lymphomas in this age group.

## Methods

### Search strategy

Medline and EMBASE were systematically searched, with no language restrictions, from inception to 1st August 2022 for the following search terms within the full text of the publication; “paediatric/pediatric”, “childhood”, “adolescent”, “diagnosis”, “clinical presentation”, “symptom”, “signs”, “lymphoma”, “Hodgkin’s”, “non-Hodgkin’s”. Reference lists of publications were further hand-searched. The full search strategies are available in the supplementary appendix (Supplementary Table 1).

### Identification of studies

Title and abstracts were screened by two researchers (DS & CZ). Any observational studies (cohort, case-control, cross-sectional) with at least 10 participants less than 20 years of age, exploring clinical presentations of any childhood and adolescent B-cell lymphoma, were eligible for full-text review. Any T-cell non-Hodgkin’s lymphomas were excluded. Due to overlap with acute lymphoblastic leukaemia, B-cell lymphoblastic lymphoma diagnoses were also excluded. All non-English studies were translated.

### Data extraction and analysis

Data was extracted using a standardised form (Supplementary Appendix Table 2). Information on publication details, number of participants and participant characteristics were included, as well as outcomes including disease site and clinical presentation.

There was variation in the description of clinical presentations across studies, with some studies reporting a cluster of symptoms (e.g. “B-symptoms”) and others reporting individual symptoms (e.g. “fever, weight loss”). B-symptoms were defined as the cluster of the following symptoms: fever, night sweats, and unintentional weight loss. We extracted the data as presented within individual studies. Different descriptions of symptoms and signs, which overall implied the same clinical presentation (e.g. difficulty breathing vs. dyspnoea) were combined for the meta-analysis. Additionally, swellings reported in narrow anatomical locations (e.g. jaw swelling and cheek swelling grouped as “head and neck swelling”) were combined for the meta-analysis. However, if a study uniquely reported a combination of symptoms or signs not present in other studies (e.g. sore throat and tonsillar mass were reported together only in one study), these were excluded from the meta-analysis.

For each study, participants with a symptom or sign, alongside site of the disease and the total number of participants in the study were recorded. Any missing data were addressed by contacting authors. For any overlapping data across studies, only the most recent and relevant publication was included. Two separate meta-analyses were carried out; the first providing pooled estimates for the prevalence of symptoms/signs reported, and the second providing pooled estimates for disease site reported. For studies reporting on symptoms and signs, proportions of study participants with symptoms in a specific disease site (e.g. abdominal pathology) were included in the systematic review but not included in the first meta-analysis as they were likely to skew results. However, if these studies reported the disease site for each study participant, they were included in the second meta-analysis.

In order to strike a balance between the need to clinically consider lymphoma as a differential diagnosis and symptoms that occur frequently in children and adolescents, only symptoms and signs occurring in more than 5% of the study population were included in the analysis. In studies reporting symptoms and signs for both Hodgkin’s and non-Hodgkin’s lymphoma, symptoms and signs were separately recorded for each type of lymphoma.

### Quality assessment

Risk of bias tool for prevalence studies based on Hoy et al. 2012 [16] was used by one researcher (DS) to assess the quality of studies. This tool contains 10 domains, of which 4 assess external validity and 6 assess internal validity. Within external validity, the tool assesses the study population’s representativeness and within internal validity, the tool assesses case definitions, data collection methodology and length of follow up. Each domain is given a high-risk or low-risk grading and subsequently each study is given an overall assessment (either high, moderate or low risk).

### Statistical analysis

Proportions from each study were inputted to determine the weighted average (pooled) proportion, through random-effects meta-analysis [17]. Briefly, we carried this out in two steps. Firstly, the proportion of each symptom or sign in an individual study was calculated and weighted by the inverse of its variance, to provide a weighted proportion. Subsequently, weighted proportions were summed and divided by the sum of the weights, to generate the pooled proportion.

Of note, as proportional data are often skewed, weighted proportions from each study were log transformed to fit a normal distribution and untransformed to provide interpretable results.

Furthermore, as between-study variation is expected across included observational studies, the pooled proportion was calculated using the restricted maximum likelihood (REML) random-effects model [17].

Heterogeneity was measured using I [2]. This measure assesses the percentage of the total observed variance, which can be accounted for by between-study variation. Small-study effect of studies was assessed using the Egger’s test [18].

An a priori decision was made to carry out the following subgroup analyses to assess heterogeneity: (1) geographical region, (2) study period, and (3) lymphoma type (Hodgkin’s vs. non-Hodgkin’s).

Our meta-analysis follows the Meta-analysis of Observational Studies in Epidemiology (MOOSE) criteria [19] and the Preferred Reporting Items for Systematic Reviews and Meta-Analyses (PRISMA) statement [20]. All analyses were carried out using the “meta” package in R. This study was registered on PROSPERO (CRD42023304949).

## Results

**Fig. 1:**
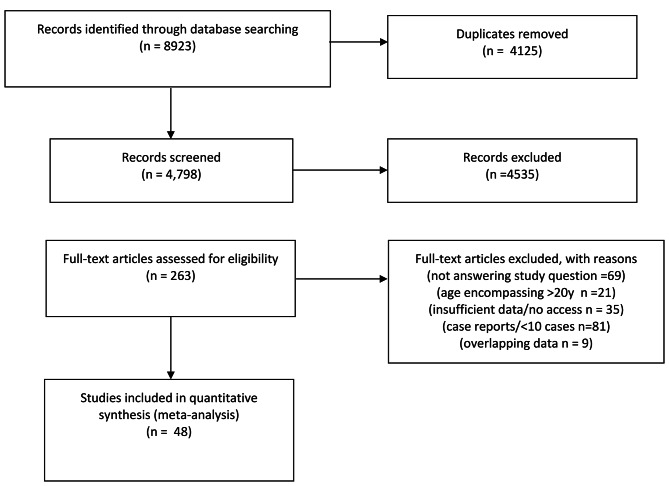
Study flow diagram

Our search strategy yielded 8923 articles, of which 263 were retrieved for full text review. 48 were eligible for meta-analysis (Fig. 1). Details of excluded studies can be found in the Supplementary Appendix (Supplementary Appendix Table 3).

Single and multi-centre studies reported on symptoms, signs and presentation sites at diagnosis of a total of 12,207 children and adolescents up to the age of 20 (Table 1, Supplementary Appendix Table 4 for quality assessment of each study). A diagnosis of lymphoma was more common in males.

**Table 1:** Demographics, quality assessment and findings of studies included within the systematic review

Study Name	Study Period	No of patients	Country	Gender (% male)	Median Age	Tumour Location	Tumour Type	Quality Assessment - Risk of Bias	Findings
Anavi et al., 1990 [21]	1976–1988	31	Israel	77	7	Head & neck	NHL	Moderate	Reports on the prevalence of 5 relevant symptoms: odontalgia, enlarged lymph node, sore throat, abnormal neurology)
Ashraf et al., 2019 [22]	2000–2012	202	Pakistan	83	9	All	HL	Moderate	Reports on prevalence B-symptoms
Bazzeh et al., 2010 [23]	1973–2005	2200	USA	-	-	All	HL	Moderate	Reports on prevalence B-symptoms
Belgaumi et al., 2008 [13]	1975–2003	368	Saudi Arabia	-	-	All	HL	Moderate	Reports on presentation site and B-symptom prevalence
Boerma et al., 2004 [24]	1994–2002	80	Netherlands	89	7	All	NHL - Burkitts	Moderate	Reports on prevalence of abdominal mass
Budiongo et al., 2015 [25]	2002–2012	63	Congo	68	9	All	NHL	Moderate	Reports on prevalence of fever, weight loss, night sweats, abdominal pain, anorexia, fatigue, lymphadenopathy, organomegaly
Burkhardt et al., 2011 [9]	1986–2007	2326	Pan European	-	-	All	NHL	Moderate	Reports on prevalence of B- symptoms
Cavdar et al., 1994 [66]	1964–1992	81	Turkey	70	5	All	NHL -Burkitts	Moderate	Reports on presentation sites
Chen et al., 2018 [26]	2011–2016	28	China	-	-	All	NHL	Moderate	Reports on prevalence of cervical mass, maxillofacial mass, organomegaly, exopthalmos
Choeyprasert et al., 2019 [27]	1998–2014	78	Thailand	75	10	All	NHL	Moderate	Reports on prevalence of symptoms by subtype of NHL
Cunha et al., 2012 [53]	1981–2007	50	Brazil	-	5	All	NHL - Burkitts	Moderate	Reports on presentation sites
Dho et al., 2018 [28]	1999–2014	302	South Korea	58	9	All	Mixed	Moderate	Reports on prevalence of abdominal pain, cough, arthralgia, sore throat, skin changes, back pain, head/neck masses
Dommett et al., 2012 [29]	1988–2010	270	UK	-	-	All	Mixed	Moderate	Reports on prevalence of head/neck mass, lymphadenopathy
Duan et al., 2016 [30]	2003–2013	83	China	82	9	All	HL	Moderate	Reports on prevalence of B- symptoms
Englund et al., 2018 [31]	1990–2010	419	Denmark/Sweden	55	-	All	HL	Moderate	Reports on prevalence of B- symptoms
Faizan et al., 2018 [32]	2012–2014	44	Pakistan	73	-	All	NHL	Moderate	Reports on prevalence of abdominal mass, lymphadenopathy, abdominal pain, head/neck mass or swelling
Ghafoor et al., 2020 [33]	2012–2018	106	Pakistan	79	8	All	HL	Moderate	Reports on prevalence of cervical lymphadenopathy, organomegaly, fever, night sweats, B-symptoms
Guo et al., 2016 [54]	2005–2013	40	China	74	8	All	NHL	Moderate	Reports on presentation site
Huang et al., 2019 [34]	2005–2017	46	Beijing, China	72	8	All	NHL - DLCL	Moderate	Reports on prevalence of B-symptoms, cervical mass, abdominal mass, abdominal pain, fever, upper respiratory tract infections
Karadeniz et al., 2007 [55]	1993–2003	61	China	80	7	All	NHL	Moderate	Reports on presentation site
Karayalcin et al., 1997 [77]	1984–1993	26	USA	85	13	All	HL	Moderate	Reports on presentation site
Karhan et al., 2019 [35]	1975–2013	102	Turkey	80	4	All	HL	Moderate	Reports on prevalence of B-symptoms
Karimi et al., 2008 [36]	1997–2002	40	Iran	-	-	All	HL	Moderate	Reports on prevalence of lymphadenopathy and fever
Katz et al., 1995	1985–1995	53	Israel	-	-	All	NHL-Burkitts	Moderate	Reports on prevalence of Bell’s palsy
Kobayashi et al., 2017 [56]	1991–2014	22	Japan	77	9	All	NHL	Moderate	Reports on presentation site
Lee et al., 2015 [37]	2000–2015	37	Singapore	77	12	Mediastinal	Mixed	Moderate	Reports on prevalence of fever, dyspnea, stridor chest pain, coughing, lymphadenopathy, night sweats, anorexia, weight loss, organomegaly, abnormal neurology, superior vena cava syndrome
Lervat et al., 2014 [57]	1989–1996	459	France	78	8	All	NHL	Moderate	Reports on presentation site
Lilja-Fishcer et al., 2018 [38]	2003–2013	30	Denmark	-	-	Head & neck	Mixed	Moderate	Reports on prevalence of swelling, fatigue, fever, pain, weight loss, dyspnoea, night sweats, lymphadenopathy, stridor, superior vena cava syndrome
Meena et al., 2019 [39]	2014–2017	26	India	81	8	All	NHL	Moderate	Reports on prevalence of abdominal mass, fever, weight loss, dyspnea, pallor, lymphadenopathy, bone pain, abdominal pain, head/neck swelling, cough, dysphagia, vomiting, edema, voice changes, diarrhoea, organomegaly, superior vena cava syndrome
Mehreen et al., 2019 [40]	2009–2015	748	Pakistan	81	-	All	HL	Moderate	Reports on prevalence of B symptom
Mlotha et al., 2011 [58]	2005–2007	661	Malawi	62	7	All	NHL - Burkitts	Moderate	Reports on presentation site
Muwakkit et al., 2004 [59]	1983–1993	42	Lebanon	79	7	All	NHL- Burkitts	Moderate	Reports on presentation site
Oliveira et al., 2020 [60]	1981–2015	110	Brazil	72	7	All	NHL	Moderate	Reports on presentation site
Orem et al., 2011 [41]	1985–2005	1217	Uganda	63	7	All	NHL -Burkitts	Moderate	Reports on prevalence of fever, weight loss, night sweats, severe infections
Otmani et al., 2008 [42}	1998–2005	37	Morocco	84	7	Head & Neck	NHL- Burkitts	Moderate	Reports on prevalence of head/neck mass, abdominal pain, nerve palsies, orbital swelling
Owusu et al., 2010 [61]	2000–2007	551	Ghana	58	7	All	NHL	Moderate	Reports on presentation site
Roh et al., 2007 [62]	2000–2005	32	Korea	69	8	Head & Neck	Mixed	Moderate	Reports on presentation site
Sandlund et al., 1997 [10]	1980–1987	92	Brazil	68	6	All	NHL-Burkitts	Moderate	Reports on presentation site
Seth et al., 2015 [43]	2005–2010	35	India	89	8	All	HL	Moderate	Reports on prevalence of lymphadenopathy and B-symptoms
Sevinir et al., 2009 [44]	-	118	Turkey	57	9	All	NHL	Moderate	Reports on prevalence of B-symptom, swelling, abdominal distension, dyspnea, cough
Sherief et al., 2015 [12]	2004–2012	142	Egypt	63	-	All	NHL	Moderate	Reports on presentation site
Sherief et al., 2015 [65]	2004–2012	59	Egypt	63	-	All	HL	Moderate	Reports prevalence of B-symptoms and presentation site
Stefan et al., 2014 [63]	1995–2010	51	South Africa	78	6	All	NHL - Burkitts	Moderate	Reports on presentation site
Trehan et al., 2013 [45]	1990–2006	206	India	91	8	All	HL	Moderate	Reports on prevalence of B- symptoms
Uccini et al., 2018 [11]	2008–2015	125	Iraq	78	6	All	NHL- Burkitts	Moderate	Reports on presentation site
Yakubu et al., 2015 [46]	1995–2009	50	Nigeria	80	-	All	Mixed	Moderate	Reports on prevalance of weight loss, jaw swelling, pallor, fever, bleeding, bone pain, lymphadenopathy, respiratory signs, organomegaly
Zhang et al., 2018 [64]	2007–2015	174	China	85	5	All	NHL- Burkitts	Moderate	Reports on presentation site
Zheng et al., 2020 [47]	2011–2017	84	China	74	6	All	NHL	Moderate	Reports on prevalence of B-symptoms

### General symptoms and signs

31 studies [9, 12, 21–48] (*n* = 9488, range per study *n* = 10 to 2326) reported on symptoms and signs for either type of B-cell lymphoma. These included cervical lymphadenopathy (48% [95% CI 20%-77%]), peripheral lymphadenopathy (51% [95% CI 37%-66%]), B-symptoms (40% [95% CI 34%-44%]), fever (43% [95% CI 34%-54%]), abdominal mass (46% [95% CI 29%-64%]), weight loss (53% [95% CI 39%-66%]), head-and-neck mass (23% [95% CI 10%-45%]), organomegaly (29% [95% CI 23%-37%]), night sweats (19% [95% CI 10%-32%]), abdominal pain (28% [95% CI 15%-47%]), bone pain (17% [95% CI 10%-28%]) and abnormal neurology (11% [95% CI 3%-28%]).

12 studies [12, 22–13, 30, 31, 33, 35, 36, 40, 43, 45] (*n* = 4678, range per study *n* = 18 to 2200) reported on the symptoms and signs for Hodgkin’s lymphoma. These included any lymphadenopathy (80% [95% CI 74%-85%]), cervical lymphadenopathy (74% [95% CI 43%-91%]), B-symptoms (40% [95% CI 33%-47%]), fever (37% [95% CI 23%-53%]) and organomegaly (22% [95% CI 15%-29%]).

**Fig. 2:**
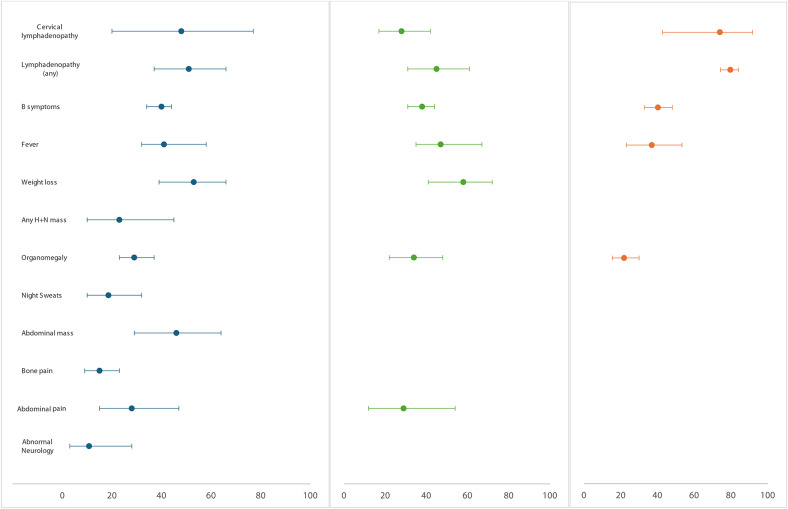
Frequency of clinical presentations associated with a lymphoma diagnosis, **i**) overall (blue), **ii**) non-Hodgkin’s (green) and **iii**) Hodgkin’s lymphoma (orange). H+N= head & neck

15 studies [9, 21, 24–27, 32, 34, 39–41, 44, 49–52] (*n* = 4258, range per study *n* = 18 to 2326) reported on the symptoms and signs for non-Hodgkin’s lymphoma. These included organomegaly (34% [95% CI 22%-48%]), B-symptoms (38% [95% CI 31%-44%]), cervical lymphadenopathy (28% [95% CI 17%-42%]), peripheral lymphadenopathy (45% [95% CI 31%-61%]), fever (47% [95% CI 35%-61%]), weight loss (58% [95% CI 41%-72%]), abdominal pain (29% [95% CI 12%-54%]) (Fig. 2).

### Presentation sites

2 studies [33, 35] (*n* = 206, range per study *n* = 102 to 106) reported on the presentation sites for Hodgkin’s lymphoma. Hodgkin’s lymphoma commonly presented with a head and neck (adenopathy) presentation (79% [95% CI 58%-91%]) and subsequently most frequently as mediastinal presentation (34% [95% CI 26%-42%]) any peripheral (adenopathy) presentation (15% [95% CI 2%-24%]), and abdominal presentation (4% [95% CI 2%-8%]).

15 studies [10, 11, 53–65] (*n* = 4161, range per study *n* = 22 to 1217) reported on the presentation sites for non-Hodgkin’s lymphoma. These were abdominal presentation (55% [95% CI 43%-68%]), head and neck presentation (16% [95% CI 8%-34%]), any peripheral lymph node presentation (4% [95% CI 1%-9%]), neurological presentation (2% [95% CI 1%-4%]), mediastinal presentation (2% [95% CI 1%-6%]), bone presentation (1% [95% CI 0.06%-3%]) and skin presentation (1% [95% CI 0.04-2%]).

**Fig. 3:**
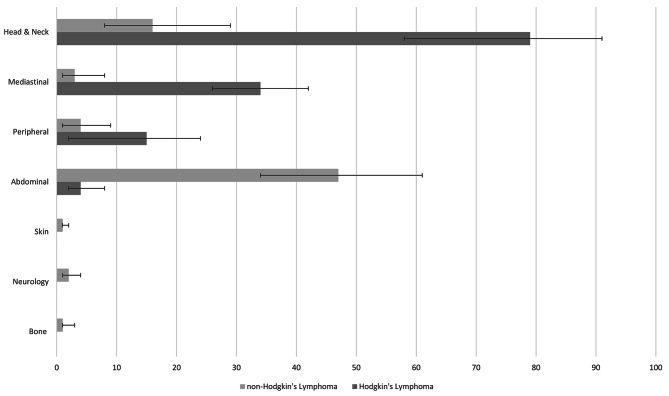
Presentation sites for Hodgkin’s (2 studies, *n*=206) and non-Hodgkin’s lymphoma (15 studies, *n*=4161)

It was possible to further characterise presentation sites according to non-Hodgkin’s lymphoma subtypes. Accordingly, 11 studies [10, 11, 24, 41, 42, 53, 58, 59, 63, 64, 66] (*n* = 3123, range per study *n* = 31 to 1217) reported on Burkitt’s lymphoma. 61% [95% CI 45%-75%] of Burkitt’s lymphoma presented with abdominal pathology, 16% [95% CI 8%-29%]) with head and neck, 4% [95% CI 1%-10%]) with peripheral lymph node pathology, and 2% [95% CI 0.07%-6%] with central nervous system (CNS) pathology. In contrast, other non-Hodgkin’s lymphomas (2 studies [67, 68], *n* = 51) were less likely to present with abdominal pathology (19% [95% CI 1%-32%]) (Fig. 3).

### Symptoms by presentation site

Four studies [21, 37, 38, 62] reported on both disease sites, the mediastinum/head-neck region, and associated symptomology, with one study focusing specifically on intensive care admissions [37]. Presentation in the head/neck or mediastinum was associated with lymphadenopathy (37% [95% CI 21%-57%]), dyspnoea (46% [95% CI 11%-86%]), fever (26% [95% CI 14%-41%]), stridor (9% [95% CI 35 − 28%]) and superior vena cava obstruction (14% [95% CI 4%-37%]).

### Heterogeneity and small study effect assessment

Heterogeneity was high across all pooled estimates of an individual clinical presentation, with I^2^ above at least 60% for each (Supplementary Appendix Table 5). Subgroup analyses were carried out for lymphoma subtype, geographical region of study and time period of publication. Region of study accounted for the majority of heterogeneity observed in the clinical features “abdominal pain”, “peripheral lymphadenopathy” and “bone pain”, whilst lymphoma subtype accounted for heterogeneity observed in “weight loss” as well as “abnormal neurology” and time period accounted for heterogeneity observed in “B-symptoms” and “abdominal mass”. We were unable to account for the heterogeneity observed in any of the other clinical presentations. (Supplementary Appendix Table 5). Small study effects were detected for the symptoms/signs “peripheral lymph node” (*p* = 0.0002) and “B-symptoms” (*p* = 0.006) (Supplementary Appendix Table 6).

## Discussion

To our knowledge this is the first systematic review and meta-analysis exploring the presenting features of B-cell lymphomas in children and adolescents. With symptoms and signs reported by 12,207 children and adolescents, this review provides a comprehensive summary of presentation patterns, highlighting the wide range of differences in presentation according to B-cell lymphoma type and location of cancer.

We found that Hodgkin’s lymphomas in this age group most commonly present in the head & neck region with lymphadenopathy, whilst non-Hodgkin’s lymphomas have much more varied presentations, with more frequent links to abdominal pathology. As B-cell non-Hodgkin’s lymphomas are most likely to have rapid progression and fast-growing masses [7], it is important to raise awareness of their varied presentation patterns within the general medical community. This may aid more prompt recognition by clinicians, faster referral to oncological services, and subsequent earlier diagnosis of these lymphomas.

We identified that B-symptoms (fever, weight loss and night sweats) were only reported in approximately 40% of presentations, which is in keeping with previous studies [7] but is often not reflected by clinical guidelines, such as the National Institute for Clinical Excellence (NICE) Suspected Cancer in the United Kingdom [69], where presence of B-symptoms are weighted significantly in suspected lymphoma diagnoses. This emphasises that although the presence of B-symptoms should be sought after when a child/adolescent presents with lymphadenopathy, other symptom combinations should also be considered. Seeking out the presence of other symptoms including abdominal pain, abdominal mass, bone pain and breathlessness, which we identified through this systematic review, may provide important information to clinicians and aid their decisions to further investigate or refer to a haemato-oncologist.

It is crucial to highlight that all studies included in this systematic review were based on secondary care/hospital data and thus, these symptoms and signs may not be fully reflective of earlier presentations to other healthcare settings, such as primary care. This is particularly important as patients are most likely to present to their primary care physician at the earliest stages of disease onset.

Furthermore, as our systematic review and meta-analysis included studies with > 10 participants, we may have not captured rarer relevant and important clinical presentations. These include gastrointestinal presentations such as intussusception [70] and bowel obstruction [71], upper respiratory tract symptoms such as asymmetrical tonsillar enlargement [72], sleep apnoea [73], wheeze and stridor [74], as well as rheumatic symptoms such as arthritis [75].

There were several limitations to this study. Firstly, there was high heterogeneity when the proportions of symptoms/signs were pooled in the meta-analysis. This is in line with reports from other meta-analysis of proportion studies [76], nevertheless, we further explored this heterogeneity through subgroup analyses in lymphoma type, geographical region, and study period, but we were unable to fully account for the observed heterogeneity in all clinical presentations. Secondly, assessment of study quality in this systematic review identified possible moderate risk of bias introduced from studies of varying quality. Finally, we detected small study effects in two symptoms, “peripheral lymph node” and “B-symptoms”, indicating that small studies estimate higher proportions for these symptoms compared to the larger studies in the meta-analysis. This implies that the estimate of prevalence for these two symptoms should be interpreted with caution.

Overall, through this systematic review and meta-analysis of proportions, we provide a summary of the prevalence of symptoms and signs associated with childhood and adolescent lymphoma. We demonstrate that well-known clinical features (lymphadenopathy and B-symptoms) are frequently observed at diagnosis but that other symptoms and signs such as abdominal pain, abdominal mass and bone pain may also play a central role in the earlier detection of lymphoma. We believe that this knowledge will increase public and clinical awareness of lymphoma presentations in children and adolescents. We also highlight that our understanding of B-cell lymphoma presentations at diagnosis in this age group is largely based on secondary care data and more primary care-based studies are needed to further characterise clinical features of lymphoma at the earlier stages of disease onset.

### Electronic supplementary material

Below is the link to the electronic supplementary material.

Supplementary Material 1

## Data Availability

No datasets were generated or analysed during the current study.
