# Supplementary material for: Presentation of B-cell lymphoma in childhood and adolescence: a systematic review and meta-analysis
Source: BMC Cancer. 2024 Jun 11;24:718. doi: 10.1186/s12885-024-12372-w (PMC11167855; doi:10.1186/s12885-024-12372-w)
Supplement: Supplementary file 1 — Supplementary Material 1 [file 12885_2024_12372_MOESM1_ESM.docx]

**Supplementary Appendix**

# ***Supplementary Table 1- Full Search Strategy- MEDLINE/EMBASE***

| 1. | p?diatr*.mp. |
| --- | --- |
| 2. | childhood.mp. or childhood/ |
| 3. | adolescent/ or adolescen*.mp. |
| 4. | child*.mp. |
| 5. | AYA.mp. |
| 6. | teenage*.mp. |
| 7. | *Young Adult/ |
| 8. | TYA.mp. |
| 9. | diagnosis/ |
| 10. | clinical presentation.mp. |
| 11. | symptom*.mp. |
| 12. | sign.mp |
| 13. | signs.mp |
| 14. | Burkitt lymphoma or nonhodgkin lymphoma or B cell lymphoma or classical Hodgkin lymphoma or lymphoma |
| 15. | 1 or 2 or 3 or 4 or 5 or 6 or 7 or 8 |
| 16. | 9 or 10 or 11 or 12 or 13 |
| 17. | 14 and 15 |
| 15. | 16 and 17 |

# **Supplementary *Table 2- Data Extraction Form (Template)***

| Study ID (PubMed) & name |  |
| --- | --- |
| Date of Publication |  |
| Contact Email of Study Author |  |
| Study Title |  |
| Study Design |  |
| Additional Information |  |
| Study Location (Country) |  |
| Diagnosis |  |
| Inclusion Criteria |  |
| Exclusion Criteria |  |
| Does the study report any significant baseline imbalances? |  |
| Does the primary paper meet the inclusion criteria for entry to the systematic review? |  |
| If no, Reason for Exclusion |  |
|  |  |
| Study Period |  |
| Study Region |  |
| Sex |  |
| Median Age |  |
| Total number of participants in study |  |
| Disease site |  |
| Tumour location |  |
| Tumour type |  |
| Stage |  |
|  |  |
| Symptom Name |  |
| Number of participants with symptom |  |
| % of participants with symptom |  |
| p-value of statistical measure |  |
| Other information |  |

- **Supplementary *Table 3 – Excluded Studies***

| *Reason for Exclusion* | *Study* |
| --- | --- |
| No analysable Data/no access | 1. [Diagnosis and treatment of anaplastic large-cell lymphoma in children and adolescents: a retrospective multicenter survey study]. Zhonghua Er Ke Za Zhi 2017; 55(3): 194-9.  2. Amitay-Laish I, Feinmesser M, Ben-Amitai D, et al. Juvenile onset of primary low-grade cutaneous B-cell lymphoma. Br J Dermatol 2009; 161(1): 140-7.  3. Arboleda LPA, Hoffmann IL, Cardinalli IA, Gallagher KPD, Santos-Silva AR, Mendonça RMH. Oral and maxillofacial cancer in pediatric patients: 30 years experience from a Brazilian reference center. Int J Pediatr Otorhinolaryngol 2020; 131: 109879.  4. Madani A, Qachouh M, Zafad S, Harif M, Benchekroun S. Childhood Hodgkin lymphoma in Casablanca Morocco: 24 years' experience in a single institution. Journal Africain du Cancer 2010; 2(1): 14-9.  5. Müller J, Csóka M, Jakab Z, Ponyi A, Erlaky H, Kovács G. [Hungarian experience with non-Hodgkin's lymphoma in childhood]. Magy Onkol 2006; 50(3): 253-9.  6. Mushtaq N, Alam MM, Aslam S, Fadoo Z, Anwar ul H. Malignant mediastinal mass in children: a single institutional experience from a developing country. JPMA - Journal of the Pakistan Medical Association 2014; 64(4): 386-9.  7. San Roman M, Aguilo F, Clapes M, et al. Burkitt's lymphoma treatment in a rural hospital in Sierra Leone. Trans R Soc Trop Med Hyg 2013; 107(10): 653-9.  8. Somjee SS, Mani S, Bufkin R, Hebert L, Velez MC, Yu LC. Pediatric Hodgkin's and non-Hodgkin's lymphomas: a retrospective analysis at the Children's Hospital of New Orleans. Journal of the Louisiana State Medical Society 2005; 157(6): 325-8.  9. Stoneham S, Ashley S, Pinkerton R, Hewitt M, Wallace WH, Shankar AG. Hodgkin's lymphoma in children aged 5 years or less - the United Kingdom experience. Eur J Cancer 2007; 43(9): 1415-21.  10. Suh JK, Gao YJ, Tang JY, et al. Clinical Characteristics and Treatment Outcomes of Pediatric Patients with Non-Hodgkin Lymphoma in East Asia. Cancer Res Treat 2020; 52(2): 359-68.  11. Tang YJ, Tang JY, Pan C, et al. [Clinical characteristics and treatment outcome of 36 cases with non-Hodgkin's lymphoma arising from mediastinum in children]. Zhonghua Er Ke Za Zhi 2009; 47(9): 687-90.  12. Vural S, Baskin D, Dogan O, et al. Diagnosis in childhood abdominal Burkitt's lymphoma. Ann Surg Oncol 2010; 17(9): 2476-9.  13. Xing PY, Shi YK, He XH, et al. [Clinical analysis of childhood and adolescent Hodgkin's lymphoma: a report of 88 cases]. Zhonghua Zhong Liu Za Zhi 2012; 34(9): 692-7.  14. Zhang YH, Duan YL, Yang J, Jin L, Zhou CJ, Gao ZF. [Clinical study of 40 children with Burkitt's and Burkitt-like lymphoma]. Zhonghua Er Ke Za Zhi 2008; 46(3): 209-14.  15. Lymphoma Study Group SGoHtSoPCMA, Lymphoma Study Group CoPCA-CA. [Diagnosis and treatment of anaplastic large-cell lymphoma in children and adolescents: a retrospective multicenter survey study]. Zhonghua Erke Zazhi 2017; 55(3): 194-9.  16. Aghai E, Hulu N, Virag I, Kende G, Ramot B. Childhood non Hodgkin's lymphoma: a study of 17 cases in Israel. Cancer 1974; 33(5): 1411-6.  17. Buchner A, Temkin D, David R. Burkitt's lymphoma in Israel. Israel Journal of Dental Medicine 1978; 27(3): 5-7, 5-7.  18. Coiffier B, Berger F, Bryon PA, Magaud JP. T-cell lymphomas: immunologic, histologic, clinical, and therapeutic analysis of 63 cases. Journal of Clinical Oncology 1988; 6(10): 1584-9.  19. Dalle JH, Mechinaud F, Michon J, et al. Testicular disease in childhood B-cell non-Hodgkin's lymphoma: the French Society of Pediatric Oncology experience. Journal of Clinical Oncology 2001; 19(9): 2397-403.  20. Franssila KO, Heiskala MK, Rapola J. Non-Hodgkin's lymphomas in childhood. A clinicopathologic and epidemiologic study in Finland. Cancer 1987; 59(10): 1837-46.  21. Gupta H, Davidoff AM, Pui CH, Shochat SJ, Sandlund JT. Clinical implications and surgical management of intussusception in pediatric patients with Burkitt lymphoma. J Pediatr Surg 2007; 42(6): 998-1001; discussion  22. Koudstaal MJ, van der Wal KG. [Early symptoms of Burkitt's lymphoma]. Nederlands Tijdschrift voor Tandheelkunde 2002; 109(1): 25-6.  23. Lee SC, Wong JE, Kueh YK. Clinical characteristics and treatment outcome of 218 patients with non-Hodgkin's lymphoma in a Singaporean institution. Singapore medical journal 2000; 41(3): 118-21.  24. Orlandi A, Sanchez AM, Calegari MA, et al. Diagnosis and management of breast lymphoma: A single-institution retrospective analysis. Translational Cancer Research 2018; 7(Supplement3): S272-S80.  25. Ostronoff M, Soussain C, Zambon E, et al. Burkitt's lymphoma in adults: A retrospective study of 46 cases. Nouvelle Revue Francaise d'Hematologie 1992; 34(5): 389-97.  26. Paryani SB, Donaldson SS, Amylon MD, Link MP. Cranial nerve involvement in children with leukemia and lymphoma. Journal of Clinical Oncology 1983; 1(9): 542-5.  27. Salem P, Anaissie E, Allam C, et al. Non-Hodgkin's lymphomas in the Middle East. A study of 417 patients with emphasis on special features. Cancer 1986; 58(5): 1162-6.  28. Sariban E, Donahue A, Magrath IT. Jaw involvement in American Burkitt's Lymphoma. Cancer 1984; 53(8): 1777-82.  29. Suvatte V, Mahasandana C, Tanphaichitr VS, Tuchinda S, Parichatikanond P, Pacharee P. Burkitt's lymphoma in Thai children: an analysis of 25 cases. Southeast Asian Journal of Tropical Medicine & Public Health 1983; 14(3): 385-93.  30. Togo B, Keita M, Medefo D, Traore F, Sidibe T. [Maxillofacial location of Burkitt's lymphoma in children treated at the University Hospital Center in Bamako, Mali: a 24-case series]. Med Trop (Mars) 2008; 68(6): 600-2.  31. Biggar RJ, Nkrumah FK, Perkins IV. Presenting clinical features of Burkitt's lymphoma in Ghana, West Africa. Journal of Tropical Pediatrics and Environmental Child Health 1979; 25(6): 157-61.  32. Duan YL, Zhang YH, Jin L, Yang J, Zhang R, Zhou CJ. [Clinical characteristics of 34 children with Hodgkin lymphoma and efficacy of treatment with chemotherapy plus low dose radiotherapy on involved sites]. Zhonghua Er Ke Za Zhi 2010; 48(9): 698-702.  33. Hausner RJ, Rosas-Uribe A, Wickstrum DA, Smith PC. Non-Hodgkin's lymphoma in the first two decades of life. A pathological study of 30 cases. Cancer 1977; 40(4): 1533-47.  34. Hutter Jr JJ, Favara BE, Nelson M, Holton CP. non Hodgkin's lymphoma in children. Correlation of CNS disease with initial presentation. Cancer 1975; 36(6): 2132-7.  35. Yang WP, Zhu CD, Gong LP, et al. Clinicopathologic and immunohistochemical study of intra-abdominal non-Hodgkin B-cell lymphoma occurring in children. [Chinese]. Chinese Journal of Pathology 2009; 38(11): 759-64. |
| *Not answering review question* | 1. Ahrensberg J, Fenger-Gron M, Vedsted P. Use of Primary Care during the Year before Childhood Cancer Diagnosis: A Nationwide Population-Based Matched Comparative Study. *PLoS ONE* 2013; **8**(3).  2. Ahrensberg JM, Fenger-Gron M, Vedsted P. Primary care use before cancer diagnosis in adolescents and young adults - A nationwide register study. *PLoS ONE* 2016; **11**(5).  3. Aricò M, Mussolin L, Carraro E, et al. Non-Hodgkin lymphoma in children with an associated inherited condition: A retrospective analysis of the Associazione Italiana Ematologia Oncologia Pediatrica (AIEOP). *Pediatr Blood Cancer* 2015; **62**(10): 1782-9.  4. Balikci HH, Gurdal MM, Ozkul MH, et al. Neck masses: diagnostic analysis of 630 cases in Turkish population. *Eur Arch Otorhinolaryngol* 2013; **270**(11): 2953-8.  5. Buecher B, Le Mentec M, Doz F, et al. [Constitutional MMR deficiency: Genetic bases and clinical implications]. *Bull Cancer* 2019; **106**(2): 162-72.  6. Cabanillas F, Rivera N, Pardo WI. Indolent Lymphomas That Present With Clinically Aggressive Features: A Subset of Low-Grade Lymphomas With a Behavior Inconsistent With the Histologic Diagnosis. *Clinical Lymphoma, Myeloma and Leukemia* 2016; **16**(10): 550-7.  7. Castano E, Glick S, Wolgast L, et al. Hypopigmented mycosis fungoides in childhood and adolescence: a long-term retrospective study. *J Cutan Pathol* 2013; **40**(11): 924-34.  8. Chen TM, Deng ZJ, Hu B, et al. [Clinical and pathological features of 13 children with Epstein-Barr virus-positive lymphoproliferative disease]. *Zhonghua Er Ke Za Zhi* 2018; **56**(10): 759-64.  9. Dang-Tan T, Trottier H, Mery LS, et al. Determinants of delays in treatment initiation in children and adolescents diagnosed with leukemia or lymphoma in Canada. *International Journal of Cancer* 2010; **126**(8): 1936-43.  10. Deosthali A, Donches K, DelVecchio M, Aronoff S. Etiologies of Pediatric Cervical Lymphadenopathy: A Systematic Review of 2687 Subjects. *Glob Pediatr Health* 2019; **6**: 2333794x19865440.  11. Dikalioti SK, Chang ET, Dessypris N, et al. Allergy-associated symptoms in relation to childhood non-Hodgkin's as contrasted to Hodgkin's lymphomas: A case-control study in Greece and meta-analysis. *European Journal of Cancer* 2012; **48**(12): 1860-6.  12. Dommett RM, Pring H, Cargill J, et al. Achieving a timely diagnosis for teenagers and young adults with cancer: the ACE "too young to get cancer?" study. *BMC Cancer* 2019; **19**(1): 616.  13. Ferry JA. Burkitt's lymphoma: clinicopathologic features and differential diagnosis. *Oncologist* 2006; **11**(4): 375-83.  14. Fragkandrea I, Nixon JA, Panagopoulou P. Signs and symptoms of childhood cancer: a guide for early recognition. *Am Fam Physician* 2013; **88**(3): 185-92.  15. Gaini RM, Romagnoli M, Sala A, Garavello W. Lymphomas of head and neck in pediatric patients. *Int J Pediatr Otorhinolaryngol* 2009; **73 Suppl 1**: S65-70.  16. Guimaraes AC, de Carvalho GM, Bento LR, Correa C, Gusmao RJ. Clinical manifestations in children with tonsillar lymphoma: A systematic review. *Critical Reviews in Oncology-Hematology* 2014; **90**(2): 146-51.  17. Guimarães AC, de Carvalho GM, Correa CR, Gusmão RJ. Association between unilateral tonsillar enlargement and lymphoma in children: A systematic review and meta-analysis. *Crit Rev Oncol Hematol* 2015; **93**(3): 304-11.  18. Gun F, Erginel B, Nvar A, Kebudi R, Salman T, Celik A. Mediastinal masses in children: Experience with 120 cases. *Pediatric Hematology and Oncology* 2012; **29**(2): 141-7.  19. Gupta V, Ambati SR, Pant P, Bhatia B. Superior vena cava syndrome in children. *Indian J Hematol Blood Transfus* 2008; **24**(1): 28-30.  20. Gupta V, Sangwaiya A, Sharma J, Kaira V, Ratan K, Sen R. Burkitt's lymphoma masquerading as intestinal obstruction: An uncommon entity with variable clinical presentation. *Clinical Cancer Investigation Journal* 2014; **3**(5): 441-3.  21. Hassan R, Klumb CE, Felisbino FE, et al. Clinical and demographic characteristics of Epstein-Barr virus-associated childhood Burkitt's lymphoma in Southeastern Brazil: epidemiological insights from an intermediate risk region. *Haematologica* 2008; **93**(5): 780-3.  22. Herbert A, Lyratzopoulos G, Whelan J, et al. Diagnostic timeliness in adolescents and young adults with cancer: a cross-sectional analysis of the BRIGHTLIGHT cohort. *The Lancet Child and Adolescent Health* 2018; **2**(3): 180-90.  23. Hochberg J, Flower A, Brugieres L, Cairo MS. NHL in adolescents and young adults: A unique population. *Pediatr Blood Cancer* 2018; **65**(8): e27073.  24. Hodak E, Amitay-Laish I, Feinmesser M, et al. Juvenile mycosis fungoides: cutaneous T-cell lymphoma with frequent follicular involvement. *J Am Acad Dermatol* 2014; **70**(6): 993-1001.  25. Holubar SD, Dozois EJ, Loftus EV, Jr., et al. Primary intestinal lymphoma in patients with inflammatory bowel disease: a descriptive series from the prebiologic therapy era. *Inflammatory Bowel Diseases* 2011; **17**(7): 1557-63.  26. Hsu CT, Chang MH, Ho MC, et al. Post-transplantation lymphoproliferative disease in pediatric liver recipients in Taiwan. *J Formos Med Assoc* 2019; **118**(11): 1537-45.  27. Huang H, Liu ZL, Zeng H, et al. Clinicopathological study of sporadic Burkitt lymphoma in children. *Chin Med J (Engl)* 2015; **128**(4): 510-4.  28. Huppmann AR, Xi L, Raffeld M, Pittaluga S, Jaffe ES. Subcutaneous panniculitis-like T-cell lymphoma in the pediatric age group: a lymphoma of low malignant potential. *Pediatr Blood Cancer* 2013; **60**(7): 1165-70.  29. Ingram L, Rivera GK, Shapiro DN. Superior vena cava syndrome associated with childhood malignancy: Analysis of 24 cases. *Medical and Pediatric Oncology* 1990; **18**(6): 476-81.  30. Karube K, Niino D, Kimura Y, Ohshima K. Classical Hodgkin lymphoma, lymphocyte depleted type: Clinicopathological analysis and prognostic comparison with other types of classical Hodgkin lymphoma. *Pathology Research and Practice* 2013; **209**(4): 201-7.  31. Nzeh DA. Atypical intrathoracic manifestations of Burkitt's lymphoma. *Pediatric Radiology* 1988; **18**(5): 411-2.  32. Pope E, Weitzman S, Ngan B, et al. Mycosis fungoides in the pediatric population: report from an international Childhood Registry of Cutaneous Lymphoma. *J Cutan Med Surg* 2010; **14**(1): 1-6.  33. Ritter J. Non-Hodgkin's lymphoma in children and adolescents. from diagnostics to follow-up. [German]. *Monatsschrift fur Kinderheilkunde* 2012; **160**(11): 1147-62.  34. Russell EC, Dunn NL, Massey GV. Lymphomas and bone tumors: clinical presentation, management, and potential late effects of current treatment strategies. *Adolescent Medicine State of the Art Reviews* 1999; **10**(3): 419-35, xi.  35. Sandlund JT, Perkins SL. Uncommon non-Hodgkin lymphomas of childhood: pathological diagnosis, clinical features and treatment approaches. *Br J Haematol* 2015; **169**(5): 631-46.  36. Sangueza M, Plaza JA. Hydroa vacciniforme-like cutaneous T-cell lymphoma: clinicopathologic and immunohistochemical study of 12 cases. *J Am Acad Dermatol* 2013; **69**(1): 112-9.  37. Sawyer R, Rosenthal DI, Maniglia AJ, Goodwin WJ. Unusual head and neck manifestations of non-Hodgkin's lymphomas in children and adults. *The Laryngoscope* 1987; **97**(10): 1136-40.  38. Schmittenbecher PP, Helmig FJ, Dressler W, Meyer U, Bertele-Harms RM. Acute multifocal gastric bleeding in non-Hodgkin-lymphoma of the upper digestive tract in a 7 year old girl. [German]. *Klinische Padiatrie* 1988; **200**(1): 45-8.  39. Sharma A, Bajpai J, Raina V, Mohanti BK. HIV-associated non-Hodgkin's lymphoma: Experience from a regional cancer center. *Indian Journal of Cancer* 2010; **47**(1): 35-9.  40. Takeda SI, Miyoshi S, Akashi A, et al. Clinical spectrum of primary mediastinal tumors: A comparison of adult and pediatric populations at a single Japanese institution. *Journal of Surgical Oncology* 2003; **83**(1): 24-30.  41. Tansel T, Onursal E, Dayloğlu E, et al. Childhood mediastinal masses in infants and children. *Turk J Pediatr* 2006; **48**(1): 8-12.  42. Vasquez L, Oscanoa M, Tello M, Tapia E, Maza I, Geronimo J. Factors associated with the latency to diagnosis of childhood cancer in Peru. *Pediatric Blood and Cancer* 2016; **63**(11): 1959-65.  43. Veneroni L, Mariani L, Lo Vullo S, et al. Symptom interval in pediatric patients with solid tumors: Adolescents are at greater risk of late diagnosis. *Pediatric Blood and Cancer* 2013; **60**(4): 605-10.  44. Wang BT, Dang HB, Wei YJ. [Clinical Efficacy and Prognostic Factors of Newly Diagnosed Children[JP] with Burkitt Lymphoma Treated by High-Dose and Short-Course Modified LMB Regimen ± Rituximab]. *Zhongguo Shi Yan Xue Ye Xue Za Zhi* 2020; **28**(2): 529-34.  45. Xu Y, Stavrides-Eid M, Baig A, et al. Quantifying treatment delays in adolescents and young adults with cancer at McGill University. *Current Oncology* 2015; **22**(6): e470-e7.  46. Ye ZJ, Zhang Z, Liang MZ, et al. Symptoms and management of children with incurable cancer in mainland China. *Eur J Oncol Nurs* 2019; **38**: 42-9.  47. Zea JM, Exelby PR, Wollner N. Abdominal non Hodgkin's lymphoma in childhood. *Journal of Pediatric Surgery* 1976; **11**(3): 363-9.  48. Ceppi F, Pope E, Ngan B, Abla O. Primary Cutaneous Lymphomas in Children and Adolescents. *Pediatric Blood and Cancer* 2016.  49. Chen CH, Wu KH, Chao YH, et al. Clinical manifestation of pediatric mediastinal tumors, a single center experience. *Medicine (United States)* 2019; **98**(32).  50. Kashif RU, Faizan M, Anwar S. Pediatric Malignant Mediastinal Masses. *J Coll Physicians Surg Pak* 2019; **29**(3): 258-62.  51. Liu T, Al-Kzayer LFY, Xie X, et al. Mediastinal lesions across the age spectrum: A clinicopathological comparison between pediatric and adult patients. *Oncotarget* 2017; **8**(35): 59845-53.  52. Malik R, Mullassery D, Kleine-Brueggeney M, et al. Anterior mediastinal masses - A multidisciplinary pathway for safe diagnostic procedures. *Journal of Pediatric Surgery* 2019; **54**(2): 251-4.  53. Pinho-Apezzato ML, Tannuri U, Tannuri AC, et al. Multiple clinical presentations of lymphoproliferative disorders in pediatric liver transplant recipients: a single-center experience. *Transplant Proc* 2010; **42**(5): 1763-8.  54. Roskin J, Diviney J, Nanduri V. Presentation of childhood cancers to a paediatric shared care unit. *Archives of Disease in Childhood* 2015; **100**(12): 1131-5.  55. Valencia Ocampo OJ, Julio L, Zapata V, et al. Mycosis Fungoides in Children and Adolescents: A Series of 23 Cases. *Actas Dermosifiliogr* 2020; **111**(2): 149-56.  56. Zagolski O, Dwivedi RC, Subramanian S, Kazi R. Non-Hodgkin's lymphoma of the sino-nasal tract in children. *J Cancer Res Ther* 2010; **6**(1): 5-10.  57. Zhu YY, Duan YT, Song LL, et al. [Clinicopathological Analysis of Children's Systemic EBV-Positive T-Cell Lymphoma]. *Zhongguo Shi Yan Xue Ye Xue Za Zhi* 2019; **27**(4): 1131-7.  58. Kaudewitz P, Stein H, Dallenbach F, et al. Primary and secondary cutaneous Ki-1+ (CD30+) anaplastic large cell lymphomas. Morphologic, immunohistologic, and clinical-characteristics. American Journal of Pathology; 135(2): 359-67.  59. Porta F, Bongiorno M, Locatelli F, et al. Kaposi's sarcoma in a child after autologous bone marrow transplantation for non-Hodgkin's lymphoma. Cancer 1991; 68(6): 1361-4.  60. Aysun S, Topcu M, Gunay M, Topaloglu H. Neurologic features as initial presentations of childhood malignancies. Pediatric Neurology 1994; 10(1): 40-3.  61. Abkari A, Nejjari N, Sbihi M, et al. Intestinal tuberculosis in children. [French]. Medecine et Maladies Infectieuses 1998; 28(8-9): 604-7.  62. Fink-Puches R, Chott A, Ardigo M, et al. The spectrum of cutaneous lymphomas in patients less than 20 years of age. Pediatric Dermatology 2004; 21(5): 525-33.  63. Stefan DC, Siemonsma F. Delay and causes of delay in the diagnosis of childhood cancer in Africa. Pediatric Blood and Cancer 2011; 56(1): 80-5.  64. Gerber N, Atoria CL, Elkin EB, Yahalom J. Characteristics and outcomes of patients with lymphocyte-predominant hodgkin lymphoma versus classical hodgkin lymphoma: A population-based analysis. International Journal of Radiation Oncology Biology Physics 2014; 1): S153-S4. |
| *Age range > 20 years* | 1. Wilder WH, Harner SG, Banks PM. Lymphoma of the nose and paranasal sinuses. Archives of Otolaryngology 1983; 109(5): 310-2.  2. Won YW, Kwon JH, Lee SI, et al. Clinical features and outcomes of Hodgkin's lymphoma in Korea: Consortium for Improving Survival of Lymphoma (CISL). Annals of Hematology 2012; 91(2): 223-33.  3. Wood L, Robinson R, Gavine L, Juritz J, Jacobs P. A single unit lymphoma experience: outcome in a Cape Town academic centre. Transfusion & Apheresis Science 2007; 37(1): 93-102.  4. Zawati I, Adouni O, Finetti P, et al. Adolescents and young adults with classical Hodgkin lymphoma in North Tunisia: Insights from an adult single-institutional study. Cancer Radiother 2020.  5. Diop S, Deme A, Dangou JM, et al. Non Hodgkin Lymphoma in Dakar: Study about 107 cases between 1986 and 1998. [French]. Bulletin de la Societe de Pathologie Exotique 2004; 97(2): 109-12.  6. Dommett RM, Redaniel MT, Stevens MCG, Hamilton W, Martin RM. Features of cancer in teenagers and young adults in primary care: a population-based nested case-control study. British Journal of Cancer 2013; 25.  7. El Weshi A, Akhtar S, Mourad WA, et al. T-cell/histiocyte-rich B-cell lymphoma: Clinical presentation, management and prognostic factors: report on 61 patients and review of literature. Leukemia & Lymphoma 2007; 48(9): 1764-73.  8. Hingorjo MR, Syed S. Presentation, staging and diagnosis of lymphoma: a clinical perspective. Journal of Ayub Medical College, Abbottabad: JAMC 2008; 20(4): 100-3.  9. Howell DA, Warburton F, Ramirez AJ, Roman E, Smith AG, Forbes LJL. Risk factors and time to symptomatic presentation in leukaemia, lymphoma and myeloma. British Journal of Cancer 2015; 113(7): 1114-20.  10. Matysiak-Budnik T, Jamet P, Fabiani B, et al. Primary intestinal B-cell lymphoma: a prospective multicentre clinical study of 91 cases. Digestive & Liver Disease 2013; 45(11): 947-52.  11. Patel J, Melly L, Sheppard MN. Primary cardiac lymphoma: B- and T-cell cases at a specialist UK centre. Annals of Oncology 2010; 21(5): 1041-5.  12. Sandhu D, Sharma A, Kumar L. Non-Hodgkin's lymphoma in Northern India: An analysis of clinical features of 241 cases. Indian Journal of Medical and Paediatric Oncology 2018; 39(1): 42-5.  13. Sandhu D, Sharma A, Kumar L, Raina V, Kochupillai V. Clinical features of non - Hodgkin lymphoma in Northern India an analysis of 241 cases. Journal International Medical Sciences Academy 2017; 30(1): 29-32.  14. Zhang J, Li M, Huang X, Liu C, Gao Z. Clinicopathological analysis of 273 cases of primary intestinal non-Hodgkin's lymphoma. [Chinese]. Zhonghua xue ye xue za zhi = Zhonghua xueyexue zazhi 2014; 35(6): 499-504.  15. Perrone T, Frizzera G, Rosai J. Mediastinal diffuse large-cell lymphoma with sclerosis. A clinicopathologic study of 60 cases. American Journal of Surgical Pathology; 10(3): 176-91.  16. Zwirner P, Grevers G, Wilmes E. Clinical manifestation of histologically classified malignant lymphomas in the ENT region. [German]. Laryngo- Rhino- Otologie 1990; 69(12): 642-6.  17. Daley MF, Partington MD, Kadan-Lottick N, Odom LF. Primary epidural Burkitt lymphoma in a child: Case presentation and literature review. Pediatric Hematology and Oncology 2003; 20(4): 333-8.  18. Chakrabarti B, Bhaduri B, Barik S, Gupta D, Chakravorty S. Primary ovarian lymphoma in a child. Journal of the Indian Medical Association 2011; 109(9): 679-80.  19. Addasi A. Age distribution of lymphoma pathology subtypes as part of haematological malignancies in Jordan: A retrospective analysis of 2653 cases in a tertiary cancer centre. Haematologica 2013; 1): 637.  20. Addasi AH. Contribution of lymphoma to the burden of haematological malignancies in Jordan: A retrospective analysis of 5153 cases in a tertiary cancer centre. Hematological Oncology 2013; 1): 214.  21. Zhao Y, Huang S, Ma C, Zhu H, Bo J. Clinical features of cardiac lymphoma: an analysis of 37 cases. Journal of International Medical Research 2021; 49(3). |
| *Case reports/ N<10 participants* | 1. Bandyopadhyay R, Sinha SK, Chatterjee U, et al. Primary pediatric gastrointestinal lymphoma. Indian Journal of Medical and Paediatric Oncology 2011; 32(2): 92-5.  2. Benoit MM, Vargas SO, Bhattacharyya N, et al. The presentation and management of mandibular tumors in the pediatric population. Laryngoscope 2013; 123(8): 2035-42.  3. Burnelli R, Fabbri E, Guerrini G, Sperlì D, D'Ambrosio A. Neurological presentation of Hodgkin lymphoma in the Italian Association of Pediatric Hematology and Oncology LH-2004 protocol. Leuk Lymphoma 2011; 52(6): 1049-54.  4. Bussell HR, Kroiss S, Tharakan SJ, Meuli M, Moehrlen U. Intussusception in children: lessons learned from intestinal lymphoma as a rare lead-point. Pediatric Surgery International 2019; 35(8): 879-85.  5. Carr TF, Lockwood L, Stevens RF, et al. Childhood B cell lymphomas arising in the mediastinum. Journal of Clinical Pathology 1993; 46(6): 513-6.  6. Chatterjee T, Gupta D, Bharadwaj R, Madan R. Burkittts Lymphoma Revisited: Series of Three Cases with Varied Clinical Presentation. Indian Journal of Hematology and Blood Transfusion 2014; 30(Supplement 1): 215-8.  7. Chen FF, Chen YP, Chen G. [Clinicopathological features and molecular genetics of paediatric-type follicular lymphoma: report of eight cases]. Zhonghua Bing Li Xue Za Zhi 2019; 48(5): 364-8.  8. Dembowska-Bagińska B, Wakulińska A, Daniluk I, et al. Non-Hodgkin lymphoma after liver and kidney transplantation in children. Experience from one center. Adv Clin Exp Med 2020; 29(2): 197-202.  9. Eades CP, Herbert SA, Edwards SG, et al. High rate of lymphoma among a UK cohort of adolescents with vertically acquired HIV-1 infection transitioning to adult care in the era of antiretroviral therapy. AIDS 2016; 30(1): 153-6.  10. Eisenbud L, Sciubba J, Mir R, Sachs SA. Oral presentations in non-Hodgkin's lymphoma: A review of thirty-one cases. Part II. Fourteen cases arising in bone. Oral Surgery Oral Medicine and Oral Pathology 1984; 57(3): 272-80.  11. Jiao G, Zheng Z, Jiang K, Zhang J, Wang B. Enteropathy-associated T-cell lymphoma presenting with gastrointestinal tract symptoms: A report of two cases and review of diagnostic challenges and clinicopathological correlation. Oncology Letters 2014; 8(1): 91-4.  12. Luria L, Nguyen J, Zhou J, et al. Manifestations of gastrointestinal plasmablastic lymphoma: a case series with literature review. World Journal of Gastroenterology 2014; 20(33): 11894-903.  13. Maazoun K, Mekki M, Sahnoun L, et al. [Intussusception owing to pathologic lead points in children: report of 27 cases]. Arch Pediatr 2007; 14(1): 4-9.  14. Nime FA, Cooper HS, Eggleston JC. Primary malignant lymphomas of the salivary glands. Cancer 1976; 37(2): 906-12.  15. Piastra M, Caresta E, Ruggiero A, Chiaretti A, Polidori G, Riccardi R. Management of critically III children with mediastinal neoplasms: A 6-year survey from a single institution. Medical and Pediatric Oncology 2003; 40(5): 329-31.  16. Piloni MJ, Molina G, Keszler A. Malignant oral-maxillary neoplasm in children and adolescents. A retrospective analysis from the biopsy service at a school of dentistry in Argentina. Acta Odontol Latinoam 2009; 22(3): 233-8.  17. Rebelo-Pontes HA, de Abreu MC, Guimaraes DM, et al. Burkitt's lymphoma of the jaws in the Amazon region of Brazil. Medicina Oral, Patologia Oral y Cirugia Bucal 2014; 19(1): e32-e7.  18. Ridgway D, Wolff LJ, Neerhout RC, Tilford DL. Unsuspected non-Hodgkin's lymphoma of the tonsils and adenoids in children. Pediatrics 1987; 79(3): 399-402.  19. Ruijun P, Na W, Yini W, Ran T, Shuo L, Zhao W. Primary lymphoma of bone: 9 cases reports and a clinical analysis of diagnosis and treatment. [Chinese]. Journal of Leukemia and Lymphoma 2015; 24(2): 101-4.  20. Saraswatula A, McShane D, Tideswell D, et al. Mediastinal masses masquerading as common respiratory conditions of childhood: A case series. European Journal of Pediatrics 2009; 168(11): 1395-9.  21. Schuh S, Wesson DE. Intussusception in children 2 years of age or older. CMAJ Canadian Medical Association Journal 1987; 136(3): 269-72.  22. Szekely G, Miltenyi Z, Mezey G, et al. Epidural malignant lymphomas of the spine: collected experiences with epidural malignant lymphomas of the spinal canal and their treatment. Spinal Cord 2008; 46(4): 278-81.  23. van Munster HE, Steur A, Hagleitner MM, Dors N. [Throat and abdominal symptoms in malignant lymphoma; recognize the alarm signals in children]. Ned Tijdschr Geneeskd 2020; 164.  24. Yoon WJ, Yoon YB, Kim YJ, Ryu JK, Kim YT. Primary pancreatic lymphoma in Korea--a single center experience. Journal of Korean Medical Science 2010; 25(4): 536-40.  25. Yu W, Wang H, Liu X, et al. Primary renal lymphoma:A report of 7 cases and literature review. [Chinese]. Chinese Journal of Clinical Oncology 2011; 38(6): 332-4+8.  26. Zombori L, Kovacs G, Csoka M, Derfalvi B. Rheumatic symptoms in childhood leukaemia and lymphoma-a ten-year retrospective study. Pediatr Rheumatol Online J 2013; 11: 20.  27. Foley RW, Aworanti OM, Gorman L, et al. Unusual childhood presentations of abdominal non-Hodgkin's lymphoma. Pediatrics International 2016; 58(4): 304-7.  28. Hong X, Khalife S, Bouhabel S, et al. Rhinologic manifestations of Burkitt Lymphoma in a pediatric population: Case series and systematic review. International Journal of Pediatric Otorhinolaryngology 2019; 121: 127-36.  29. Nagasaka T, Nakamura S, Medeiros LJ, Juco J, Lai R. Anaplastic large cell lymphomas presented as bone lesions: A clinicopathologic study of six cases and review of the literature. Modern Pathology 2000; 13(10): 1143-9.  30. Perwein T, Lackner H, Ebetsberger-Dachs G, et al. Management of children and adolescents with gray zone lymphoma: A case series. Pediatric Blood and Cancer 2020; 67(5).  31. Teh CSL, Jayalakshmi P, Chong SYC. Waldeyer ring lymphoma: A case series. Ear, Nose and Throat Journal 2014; 93(9): E22.  32. Tseng WY, Li YW, Su IJ, Lin DT, Huang KM. Burkitt's and non-Burkitt's type lymphoma: clinicopathological and radiological manifestations. Journal of the Formosan Medical Association 1991; 90(4): 357-64.  33. Urasinski T, Kamienska E, Gawlikowska-Sroka A, et al. Pediatric pulmonary Hodgkin lymphoma: analysis of 10 years data from a single center. Eur J Med Res 2010; 15 Suppl 2(Suppl 2): 206-10.  34. Yang H, Yang Z, Sang X, et al. Diagnosis and treatment of primary hepatic lymphoma. Translational Cancer Research 2018; 7(3): 720-8.  35. AlSemari MA, Maktabi A, AlSamnan MS, Alrajeh MS, Strianese D. Conjunctival Pediatric Follicular Lymphoma: Case Report and Literature Review. Ophthalmic Plastic & Reconstructive Surgery; 36(1): e14-e5.  36. Beatty C, Okal R, Lynch MC. Cutaneous Langerhans Cell Histiocytosis as Presenting Sign of Systemic B-Cell Lymphoma. American Journal of Dermatopathology; 43(12): 990-2.  37. Duan L, Liu J, Zhang Y, et al. Primary Pituitary Lymphoma in Immunocompetent Patients: A Report on Two Case Studies and the Review of Literature. Frontiers in Endocrinology; 11: 562850.  38. Dzoljic E, Stosic-Opincal T, Skender-Gazibara M, et al. Primary lymphoma of the brain in a young man whose brother died of hemophagocytic lymphohistiocytosis: case report. Srpski Arhiv Za Celokupno Lekarstvo; 143(1-2): 63-7.  39. Esptein LG, DiCarlo FJ, Jr., Joshi VV, et al. Primary lymphoma of the central nervous system in children with acquired immunodeficiency syndrome. Pediatrics; 82(3): 355-63.  40. Gopal M, Fisher R. A case report of B-cell lymphoma masquerading as superior mesenteric artery syndrome. Journal of Pediatric Surgery; 42(11): 1926-7.  41. Liu RS, Liu HC, Bu JQ, Dong SN. Burkitt's lymphoma presenting with jaw lesions. Journal of Periodontology; 71(4): 646-9.  42. Masszi I, Kisida E, Bely M. [Acute abdominal signs of B-cell non-Hodgkin lymphoma at a young age]. Magyar Sebeszet; 53(2): 76-8.  43. Quadri SA, Sobani ZA, Enam SA, Enam K, Ashraf MS. Primary central nervous system lymphoma causing multiple spinal cord compression and carcinomatous meningitis in a 6-year-old: a case report. Journal of Pediatric Hematology/Oncology; 33(4): 312-5.  44. Santilli V, Mora N, Aquilani A, et al. Burkitt's lymphoma mimicking EBV disease as first sign of vertical HIV infection in an adolescent. Italian Journal of Pediatrics; 36: 34.  45. Toader C, Toader M, Stoica A, et al. Tonsillar lymphoma masquerading as obstructive sleep apnea - pediatric case report. Romanian Journal of Morphology & Embryology; 57(2 Suppl): 885-91.  46. Vandenberghe E, Van Hove J, Brock P, et al. Non-endemic Burkitt's lymphoma in a patient with Bloom's syndrome. Leukemia & Lymphoma; 10(4-5): 377-82.  47. Vigier S, Nicollas R, Roman S, Barlogis V, Coulibaly B, Triglia JM. [Burkitt's leukemia presenting as atypical acute mastoiditis in a 7-month-old child]. Archives de Pediatrie; 20(12): 1317-20.  48. Vofo BN, Ngankam GVF, Ngwasiri CA, Atem JA, Aminde LN. Acute blindness as a presenting sign of childhood endemic Burkitt's lymphoma in Cameroon: a case report. Journal of Medical Case Reports [Electronic Resource]; 12(1): 129.  49. Wu X, Zhou C, Jin L, Liu H, Liu J, Zhao S. Primary pulmonary lymphoma in children. Orphanet Journal Of Rare Diseases; 14(1): 35.  50. Shende A, Lipsitz P, Catalano L, Gandhi M, Shenker IR, Gauthier B. Lymphoma presenting as a suppurating inguinal mass. Children's Hospital Quarterly 1995; 6(3): 167-8.  51. Vecsei A, Attarbaschi A, Krammer U, Mann G, Gadner H. Pruritus in pediatric non-Hodgkin's lymphoma. Leukemia and Lymphoma 2002; 43(9): 1885-7.  52. Daley MF, Partington MD, Kadan-Lottick N, Odom LF. Primary epidural Burkitt lymphoma in a child: Case presentation and literature review. Pediatric Hematology and Oncology 2003; 20(4): 333-8.  53. Mitsukawa N, Nagata T, Imamura Y, Ishikawa T, Hosaka Y. Primary Malignant Lymphoma of the Parotid Gland in a Young Patient; A Case Report. [Japanese]. Japanese Journal of Plastic and Reconstructive Surgery 2004; 47(3): 289-94.  54. Brankov O, Dumanov K, Stoilov S, Doinova P, Drebov R, Khristozova I. Non-Hodgkin's primary intestinal lymphoma - a cause of acute abdominal manifestation in children. [Bulgarian]. Khirurgiia 2007; (4): 10-3.  55. La Barba G, Sau A, Onofrillo D, et al. Hodgkin lymphoma: Polyarthritis as unusual presenting feature in a pediatric patient. Haematologica 2009; 4): 214.  56. Demirkaya M, Sevinir B, Ozdemir O, Nazlioglu HO, Okan M. Lymphoma of the Cavernous Sinus Mimicking Tolosa-Hunt Syndrome in a Child. Pediatric Neurology 2010; 42(5): 351-4.  57. Chakrabarti B, Bhaduri B, Barik S, Gupta D, Chakravorty S. Primary ovarian lymphoma in a child. Journal of the Indian Medical Association 2011; 109(9): 679-80.  58. Zengi O, Zengi S, Yitit O. A lymphoma case without any finding of physical examination except jaundice. [Turkish, English]. Turkish Journal of Biochemistry Conference: 24th National Biochemistry Congress Konya Turkey Conference Publication: 2012; 37(SPEC. ISS. 1).  59. Kourti M, Mantadakis E, Anastasiou A, Koliouskas D. Acute visual loss as the initial presentation in a child with systemic Burkitt lymphoma. Indian Journal of Medical and Paediatric Oncology 2013; 34(2): 99-100.  60. Camero A, Mathur N, Sostre C. A not so starry night: Atypical duodenal burkitt's lymphoma in a patient with celiac disease and chronic hepatitis C. American Journal of Gastroenterology 2014; 2): S317.  61. Hall N. Sarcoidosis-lymphoma syndrome in a pediatric patient. Pediatric Blood and Cancer 2014; 1): S25.  62. Dror T, Donovan V, Strubel N, Bhaumik S. Sporadic burkitt lymphoma presenting with sphenoid bone invasion and acute pancreatitis in a child. Pediatric Blood and Cancer Conference 2020; 67(SUPPL 2).  63. Siddiqi A. A Young Girl with Fever That Won't Go Away. Annals of Allergy, Asthma and Immunology 2020; 125(5 Supplement): S87.  64. Bandyopadhyay R, Bandyopadhyay SK, Dhua D, Roy S. Primary cutaneous precursor B-cell lymphoblastic lymphoma with late dissemination. Singapore Medical Journal 2011; 52(12): e258-e61.  65. Buchner A, Temkin D, David R. Burkitt's lymphoma in Israel. Israel Journal of Dental Medicine 1978; 27(3): 5-7, 5-7.  66. Camilo GB, Machado DC, de Oliveira CE, et al. Burkitt lymphoma with initial clinical presentation due to infiltration of the central nervous system and eye orbits. American Journal of Case Reports 2014; 15: 404-10.  67. Ekanayake CD, Punchihewa R, Wijesinghe PS. An atypical presentation of an ovarian lymphoma: A case report 11 Medical and Health Sciences 1114 Paediatrics and Reproductive Medicine 11 Medical and Health Sciences 1112 Oncology and Carcinogenesis. Journal of Medical Case Reports 2018; 12(1).  68. Fang YH, Peng KR, Chen FB, Tang LJ, Chen J. [Pancreatitis as the initial manifestation and abdominal lymph node enlargement in a boy]. Zhongguo Dangdai Erke Zazhi 2018; 20(10): 844-7.  69. Grasso D, Borreggine C, Ladogana S, et al. Sporadic Burkitt's lymphoma/acute B-cell leukaemia presenting with progressive proptosis and orbital mass in a child. Neuroradiology Journal 2016; 29(3): 231-5.  70. Grewal JS, Gunaratnam NT, Krauss JC, Smith LB. Unusual case of Burkitt lymphoma with thyroid gland and abdominal involvement. American Journal of Case Reports 2010; 11: 16-9.  71. Guimaraes AC, de Carvalho GM, Gusmao RJ. Tonsillar lymphoma in children with unilateral tonsillar enlargement. Revista Paulista de Pediatria 2012; 30(2): 288-91.  72. Gupta V, Sangwaiya A, Sharma J, Kaira V, Ratan K, Sen R. Burkitt's lymphoma masquerading as intestinal obstruction: An uncommon entity with variable clinical presentation. Clinical Cancer Investigation Journal 2014; 3(5): 441-3.  73. Jiao G, Zheng Z, Jiang K, Zhang J, Wang B. Enteropathy-associated T-cell lymphoma presenting with gastrointestinal tract symptoms: A report of two cases and review of diagnostic challenges and clinicopathological correlation. Oncology Letters 2014; 8(1): 91-4.  74. Jones GR, Mason WH, Fishman LS, DeClerck YA. Primary central nervous system lymphoma without intracranial mass in a child. Diagnosis by documentation of monoclonality. Cancer 1985; 56(12): 2804-8.  75. Kuo TT, Yang CP, Lin CH, Chang CH. Lymphoblastic lymphoma presenting as a huge intracavitary cardiac tumor causing heart failure. Pediatric pathology / affiliated with the International Paediatric Pathology Association 1987; 7(3): 341-9.  76. Madabhavi I, Patel A, Revannasiddaiah S, et al. Primary esophageal burkitt's lymphoma: A rare case report and review of literature. Gastroenterology and Hepatology from Bed to Bench 2014; 7(4): 230-7.  77. Romay Ageitos A, Freire Bruno J, Lopez Vzquez AM, Castro Lopez I, Pavn Freire A. Bilateral primary renal Burkitt lymphoma presenting with acute renal failure. [Spanish]. Anales de Pediatria 2010; 73(4): 199-201.  78. Stepan L, Shaw CKL. Lymphoma of the sphenoid sinus presenting as abducens nerve palsy in a child. Surgical Practice 2018; 22(3): 138-40.  79. Svoboda WE, Aaron GR, Albano EA. North American Burkitt's lymphoma presenting with intraoral symptoms. Pediatric Dentistry 1991; 13(1): 52-8.  80. Wamalwa A, Siwo EA, Mburugu PM, Mohamed M. Chronic ileocecal intussusception secondary to non-hodgkins lymphoma. Annals of African Surgery 2015; 12(1): 52-5.  81. Teh CSL, Jayalakshmi P, Chong SYC. Waldeyer ring lymphoma: A case series. Ear, Nose and Throat Journal 2014; 93(9): E22. |
| *More recent/relevant for study population* | 1. Cavdar AO, Gozdasoglu S, Yavuz G, et al. Burkitt's lymphoma between African and American types in Turkish children: clinical, viral (EBV), and molecular studies. Medical & Pediatric Oncology 1993; 21(1): 36-42.  2. Mava Y, Baba UA, Timothy SY, Pius S, Ambe JP. Retrospective study of childhood burkitts lymphoma in north eastern Nigeria. West African Journal of Medicine 2013; 32(4): 297-301.  3. Stefan DC, Stones D, Dippenaar A, Kidd M. Ethnicity and characteristics of Hodgkin lymphoma in children. Pediatr Blood Cancer 2009; 52(2): 182-5.  4. Englund A, Hopstadius C, Enblad G, Gustafsson G, Ljungman G. Hodgkin lymphoma-a survey of children and adolescents treated in Sweden 1985-2009. Acta Oncologica 2015; 54(1): 41-8.  5. Faizan M, Taj MM, Anwar S, et al. Comparison of Presentation and Outcome in 100 Pediatric Hodgkin Lymphoma Patients Treated at Children Hospital, Lahore, Pakistan and Royal Marsden Hospital, UK. J Coll Physicians Surg Pak 2016; 26(11): 904-7.  6. Sherief LM, Elsafy UR, Abdelkhalek ER, Kamal NM, Youssef DM, Elbehedy R. Disease patterns of pediatric non-Hodgkin lymphoma: A study from a developing area in Egypt. Mol Clin Oncol 2015; 3(1): 139-44.  7. Stefan DC, Stones D, Newton R. Burkitt lymphoma in South African children: one or two entities? Transfusion & Apheresis Science 2011; 44(2): 191-4. |

- **Supplementary Table 4– Quality assessment of prevalence studies using Hoy et al., 2012**

| **Study Name** | **Close representation to the national population** | **Appropriate sample of target population** | **Random selection process** | **Non-response bias** | **Data collection – from participant** | **Appropriate case definition** | **Valid measurement tool** | **Data collection -mode** | **Appropriate prevalence period** | **Appropriate denominator/numerator** | **Overall** |
| --- | --- | --- | --- | --- | --- | --- | --- | --- | --- | --- | --- |
| Anavi et al., 1990^1^ | H | H | L | L | H | L | H | L | L | L | M |
| Ashraf et al., 2019^2^ | H | H | L | L | H | L | H | L | L | L | M |
| Atas et el., 2014^3^ | H | H | H | L | H | L | H | L | L | L | M |
| Bazzeh et al, 2010 ^4^ | L | L | L | L | H | L | H | L | L | L | M |
| Belgaumi et al., 2008 ^5^ | H | H | H | L | H | L | H | L | L | L | M |
| Boerma et al., 2004^6^ | L | L | L | L | H | L | H | L | L | L | M |
| Budiongo et al., 2015^7^ | H | H | L | L | H | L | H | L | L | L | M |
| Burkhardt et al., 2011^8^ | L | L | L | L | H | L | H | L | L | L | M |
| Cavdar et al., 1994^9^ | H | H | H | L | H | L | H | L | L | L | M |
| Chen et al., 2018^10^ | H | H | H | L | H | L | H | L | L | L | M |
| Choeyprasert et al., 2019^11^ | H | H | H | L | H | L | H | L | L | L | M |
| Cunha et al., 2012^12^ | H | H | H | L | H | L | H | L | L | L | M |
| Dho et al., 2018^13^ | H | H | H | L | H | L | H | L | L | L | M |
| Dommett et al., 2013^14^ | L | L | L | L | H | L | H | L | L | L | M |
| Duan et al., 2016^15^ | H | H | H | L | H | L | H | L | L | L | M |
| Englund et al., 2018^16^ | L | L | L | L | H | L | H | L | L | L | M |
| Faizan et al., 2018^17^ | H | H | L | L | H | L | H | L | L | L | M |
| Ghaffoor et al., 2020^18^ | H | H | L | L | H | L | H | L | L | L | M |
| Guo et al., 2016^19^ | H | H | H | L | H | L | H | L | L | L | M |
| Huang et al., 2019^20^ | H | H | H | L | H | L | H | L | L | L | M |
| Karadeniz et al., 2007^21^ | H | H | H | L | H | L | H | L | L | L | M |
| Karayalcin et al., 1997^22^ | L | L | L | L | H | L | H | L | L | L | M |
| Karhan et al., 2019^23^ | H | H | H | L | H | L | H | L | L | L | M |
| Karimi et al., 2008^24^ | H | H | H | L | H | L | H | H | L | L | M |
| Katz et al., 1995 | H | H | H | L | H | L | H | H | L | L | M |
| Kobayashi et al., 2017^25^ | L | L | H | L | H | L | H | L | L | L | M |
| Lee et al., 2015^26^ | H | H | H | L | H | L | H | L | L | L | M |
| Lervat et al., 2014^27^ | L | L | L | L | H | L | H | L | L | L | M |
| Lilja-Fishcer et al., 2018^28^ | H | H | L | L | H | L | H | L | L | L | M |
| Meena et al., 2019^29^ | H | H | L | L | H | L | H | H | L | L | M |
| Mehreen et al., 2019^30^ | H | H | H | H | H | L | H | H | L | L | M |
| Mlotha et al., 2011^31^ | H | H | H | L | H | L | H | L | L | L | M |
| Muwakkit et al., 2004^32^ | H | H | L | L | H | L | H | H | L | L | M |
| Oliviera et al., 2020^33^ | H | H | L | L | H | L | H | L | L | L | M |
| Orem et al., 2011^34^ | L | L | L | L | H | L | H | L | L | L | M |
| Otmani et al., 2008^35^ | H | H | L | L | H | L | H | H | L | L | M |
| Owusu et al., 2010^36^ | H | H | H | L | H | L | H | H | L | L | M |
| Roh et al., 2007^37^ | H | H | L | L | H | L | H | H | L | L | M |
| Sandlund et al., 1997^38^ | H | H | L | L | H | L | H | L | L | L | M |
| Seth et al., 2015^39^ | H | H | H | L | H | L | H | H | L | L | M |
| Sevinir et al., 2009^40^ | H | H | H | L | H | L | H | H | L | L | M |
| Sherief et al., 2015^41^ | H | L | L | L | H | L | H | H | L | L | M |
| Sherief et al., 2015^42^ | H | L | L | L | H | L | H | H | L | L | M |
| Stefan et al., 2014^43^ | H | H | H | L | H | L | H | H | L | L | M |
| Trehan et al., 2013^44^ | H | H | H | L | H | L | H | H | L | L | M |
| Uccini et al., 2018^45^ | H | H | H | L | H | L | H | H | L | L | M |
| Yakubu et al., 2015^46^ | H | H | H | L | H | L | L | L | L | L | M |
| Zhang et al., 2018^47^ | H | H | H | L | H | L | H | H | L | L | M |
| Zheng et al., 2020^48^ | H | H | H | L | H | L | H | H | L | L | M |

- **Supplementary Table 5- Assessment of heterogeneity through subgroup analysis of i) lymphoma type, ii) time period of study and iii) region of study**

| Symptom & Subgroup | I^2^ (%, heterogeneity before subgroup analysis) | Coefficient (95%CI) | p-value | R2 (% heterogeneity accounted for) |
| --- | --- | --- | --- | --- |
| **Peripheral lymph node** | | | | |
| Lymphoma Type | 87 | 1.18 (-0.34 to 2.71) | 0.1 | 13.6 |
| Time period of study |  | -0.75 (-2.03 to 0.54) | 0.3 | 6.5 |
| Region of study |  | 2.43 (0.98 to 3.89) | 0.01 | 71.2* |
| **Cervical lymph node** | | | | |
| Lymphoma Type | 94 | -1.79 (-3.83 to 0.25) | 0.1 | 24.2 |
| Time period of study |  | 0.28 (-1.53 to 2.09) | 0.8 | 0 |
| Region of study |  | -3.07 (-6.59 to 0.45) | 0.1 | 17.9 |
| **Weight loss** | | | | |
| Lymphoma Type | 80 | -1.7 (2.92 to -0.48) | 0.006 | 49.3* |
| Time period of study |  | 0.23 (-1.29 to 1.76) | 0.8 | 0 |
| Region of study |  | 1.77 (0.05 to 3.48) | 0.05 | 32.9 |
| **Abdominal mass** | | | | |
| Lymphoma Type | 85 | 0.63 (-1.45 to 2.72) | 0.6 | 0 |
| Time period of study |  | -1.23 (-2.4 to -0.2) | 0.02 | 53.7* |
| Region of study |  | -1.59 (-3.4 to 0.27) | 0.1 | 13.5 |
| **B-symptoms** | | | | |
| Lymphoma Type | 92 | 0.25 (-0.29 to 0.78) | 0.4 | 0 |
| Time period of study |  | -0.44 (-0.72 to -0.11) | 0.008 | 28.6* |
| Region of study |  | -0.65 (-2.17 to 0.87) | 0.4 | 0 |
| **Fever** | | | | |
| Lymphoma Type | 80 | 0.17 (-0.69 to 1.03) | 0.7 | 5.2 |
| Time period of study |  | 0.39 (-0.16 to 0.95) | 0.2 | 8.4 |
| Region of study |  | -0.86 (-1.77 to 0.04) | 0.06 | 27.9 |
| **Organomegaly** | | | | |
| Lymphoma Type | 62 | 0.57 (-0.52 to 1.66) | 0.3 | 5.2 |
| Time period of study |  | 0.26 (-0.60 to 1.12) | 0.6 | 0 |
| Region of study |  | 0.52 (-0.85 to 1.89) | 0.5 | 0 |
| **Abdominal pain** | | | | |
| Lymphoma Type | 88 | 0.21 (-2.15 to 2.56) | 0.8 | 0 |
| Time period of study |  | 0.85 (-0.91 to 2.62) | 0.3 | 0 |
| Region of study |  | -2.1 (-2.8 to -1.35) | <0.001 | 100* |
| **Any head/neck mass** | | | | |
| Lymphoma Type | 91 | 1.46 (-0.21 to 3.14) | 0.1 | 39.4 |
| Time period of study |  | 0.18 (-1.05 to 1.42) | 0.8 | 0 |
| Region of study |  | -0.79 (-2.8 to 1.22) | 0.4 | 25.3 |
| **Night sweats** | | | | |
| Lymphoma Type | 76 | -1.25 (-2.8 to 0.32) | 0.1 | 25.8 |
| Time period of study |  | 0.79 (-1.57 to 2.97) | 0.5 | 0 |
| Region of study |  | 0.97 (-2.18 to 4.1) | 0.6 | 0 |
| **Bone pain** | | | | |
| Lymphoma Type | 57 | 0.51 (-0.7 to 1.72) | 0.4 | 11.8 |
| Time period of study |  | -0.35 (-1.86 to 1.15) | 0.6 | 0 |
| Region of study |  | -1.03 (-1.97 to -0.09) | 0.03 | 72.2* |
| **Abnormal neurology** | | | | |
| Lymphoma Type | 90 | 3.11 (2.26 to 3.96) | <0.001 | 100* |
| Time period of study |  | 0.34 (-2.34 to 3.02) | 0.8 | 0 |
| Region of study |  | -0.06 (-4.04 to 3.91) | 0.2 | 27.1 |

- **Supplementary Table 6– Assessment of small study effect using Egger’s Test.**

| **Symptom & Subgroup** | **z-value** | **p-value** |
| --- | --- | --- |
| Peripheral lymph node | 3.76 | 0.0002 |
| Cervical lymph node | 1.21 | 0.21 |
| Weight loss | -0.87 | 0.38 |
| Abdominal mass | 2.7 | 0.006 |
| B-symptoms | 0.77 | 0.44 |
| Fever | -0.24 | 0.81 |
| Organomegaly | -0.38 | 0.70 |
| Abdominal pain | -0.04 | 0.96 |
| Any head/neck mass | 0.85 | 0.39 |
| Night sweats | -1.9 | 0.06 |
| Bone pain | 1.84 | 0.07 |
| Abnormal neurology | -0.62 | 0.34 |
